# Challenges and opportunities for regional collaboration for strategic purchasing in Southeast Asia

**DOI:** 10.1016/j.lansea.2023.100227

**Published:** 2023-05-31

**Authors:** Capucine Barcellona, Swee Kheng Khor, Jeremy Lim

**Affiliations:** Saw Swee Hock School of Public Health, National University of Singapore, Singapore

Southeast Asia (SEA) has seen an increase in healthcare spending in recent decades,^1^ but healthcare needs remain high^2^ and measures of population health remain below average.^3^ There is a growing recognition that more money alone cannot achieve UHC; how funds are spent is an equally important element of health financing. While higher expenditure is generally related to better health outcomes,^4^ some countries have achieved good outcomes even with constrained resources thanks to their effective use of funds.^5^ Indeed, national health expenditure (% GDP) is not always correlated to a higher UHC Index Score.

Strategic purchasing has been advocated as an important tool to effectively allocate health resources, free up fiscal space, and improve health outcomes.^6^ In SEA—where government procurement is significant yet not entirely efficient—strategic purchasing will be important to enable progress toward Universal Health Coverage (UHC).

**Figure:**
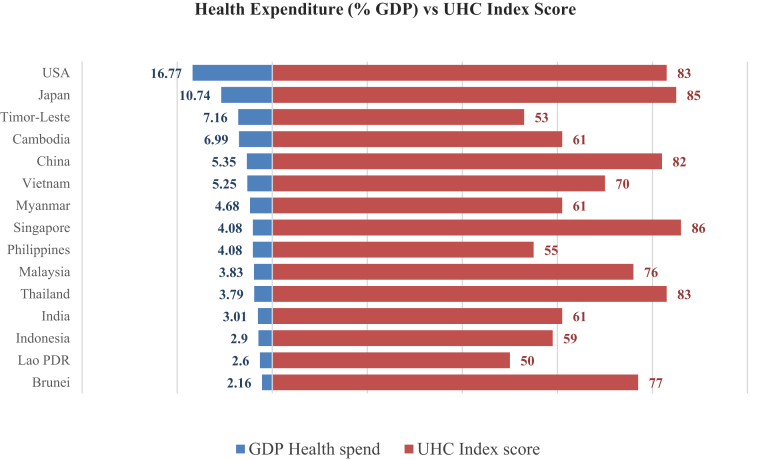

“Strategic purchasing”—defined as the efficient allocation of funds for healthcare services and goods to achieve health system goals—is driven by information on a country's health needs, provider performance, and cost-effectiveness assessments among other data. Strategic purchasing involves deciding what goods and services to buy; selecting the providers from whom to buy; and deciding how to buy by setting contract terms, payment mechanisms, and provider incentives. Reforms to make healthcare purchasing more strategic (e.g. benefit specification and performance monitoring) have already contributed to improved care quality, access, and financial protection in Kenya^7^ and Nigeria.^8^ Southeast Asian countries can likewise benefit.

While several international consortia have focused on strategic purchasing—such as RESYST (Resilient and Responsive Health Systems) and SPARC (Strategic Purchasing for Africa Resource Centre)—no Southeast Asia-focused strategic purchasing network currently exists. The region lacks cross-country learning opportunities and high-level assessments of its healthcare financing systems. The Southeast Asia Regional Collaborative for Health (SEARCH), housed at the Saw Swee Hock School of Public Health, National University of Singapore, initiated research in 2022 to review the health purchasing mechanisms of all ASEAN countries and provides national and regional-level policy recommendations, supported by six regional Country Convenors.

Initial research shows three main themes. First, there is a high diversity of financing and purchasing mechanisms across Southeast Asia existing within equally diverse health systems and forms of government. Thailand, Vietnam, and Indonesia, among others, have a purchaser-provider split whereby the principal purchaser is separate from healthcare providers and has autonomy over fund distribution and contracting. Other countries, like Brunei and Malaysia, integrate the purchaser-provider function in their respective Ministries of Health.

Second, Southeast Asia's strategic purchasing capabilities are in the early stages. Innovators like Indonesia and the Philippines are testing new ways of purchasing—for example, scaling up the *Jaminan Kesehatan Nasional* national health insurance scheme in Indonesia and transforming provider payment mechanisms under PhilHealth, the national purchaser of health services in the Philippines. Lower-income countries like Lao PDR are also implementing health financing reforms which could lay a foundation for future strategic purchasing.

Third, Southeast Asia lacks the health and financing data necessary to enable strategic purchasing. Most SEA health systems are characterised by data fragmentation and non-interoperability.^9^ Even when health data systems are in place, these may not capture information relevant to strategic purchasing. For example, Lao PDR's 2014 implementation of DHIS2 has increased data availability, but this is not used to inform purchasing decisions.

There are various challenges to a wide regional implementation of strategic purchasing. The lack of data and IT capabilities in Southeast Asia will hinder evidence-based purchasing decisions, and governments’ abilities evaluate the outcomes of such decisions on health systems once they are made. A majority of Southeast Asian countries also lack the human capacity to collect relevant data at health facilities due to technical barriers and skilled staff. Finally, regulatory capacity to govern health purchasing—for example, legislation defining clear mandates for purchasers—is also limited, as are continuous monitoring and enforcement capabilities.

Our preliminary analysis shows three opportunities to improve strategic purchasing in Southeast Asia that SEARCH intends to pursue. First, collaborative learning networks can bring together policymakers, purchasers, and providers to identify best practices and common challenges. This could potentially mitigate the effects of having highly diverse health systems in the region. Second, the post-COVID emphasis on health systems resilience can spotlight strategic purchasing as an effective financing tool to SEA policymakers. Third, capacity-building efforts—including policy-relevant recommendations contextualised to individual health systems—can enhance governments’ preparedness for strategic purchasing.

Leveraging these opportunities will help countries overcome their roadblocks to strategic purchasing by enhancing their data collection, IT systems, and human capacity. Our work will test the hypothesis that knowledge-sharing consortia can enhance strategic purchasing and that this, in turn, will be an effective tool to improve health outcomes and unlock UHC.

Southeast Asia has demonstrated increasing commitment to UHC and health financing reform. Strategic purchasing should not be overlooked as an enabler of cost-efficient, equitable health systems. Regional collaboration for strategic purchasing is necessary for governments to manage a world of increasing inflation and rising healthcare costs.

## Contributors

CB and SKK conceptualised and designed the draft. CB wrote the first draft. SKK and JL reviewed and edited the manuscript. All authors approved the manuscript for submission.

## Ethics approval

Research for this project was granted approval from the National University of Singapore Institutional Review Board.

## Declaration of interests

No conflicts to declare.
